# Topological expansion of Boehm’s brushes via structured light

**DOI:** 10.1073/pnas.2532243123

**Published:** 2026-07-09

**Authors:** Dmitry A. Pushin, Iman Salehi, Amy Chow, Andrew E. Silva, Pinki Chahal, David G. Cory, Mukhit Kulmaganbetov, Gary P. Misson, Naume Shentevski, Taranjit Singh, Shelby E. Temple, Benjamin Thompson, Dusan Sarenac

**Affiliations:** ^a^ Institute for Quantum Computing, University of Waterloo, Waterloo, ON N2L3G1, Canada; ^b^ Department of Physics and Astronomy, University of Waterloo, Waterloo, ON N2L3G1, Canada; ^c^ School of Optometry and Vision Science, University of Waterloo, Waterloo, ON N2L3G1, Canada; ^d^Incoherent Vision Inc., Wellesley, ON N0B2T0, Canada; ^e^Centre for Eye and Vision Research, Shatin, Hong Kong; ^f^ Department of Psychology, Idaho State University, Pocatello, ID 83209; ^g^ Department of Physics, University at Buffalo, State University of New York, Buffalo, NY 14260; ^h^ Department of Chemistry, University of Waterloo, Waterloo, ON N2L3G1, Canada; ^i^ School of Optometry, Aston University, Birmingham B4 7ET, United Kingdom; ^j^ Division of Research and Innovation, University of Bristol, Bristol BS8 1QU, United Kingdom; ^k^Azul Optics Ltd., Henleaze, Bristol BS9 4QG, United Kingdom

**Keywords:** structured light, entoptic phenomena, vision science, spin–orbit beams, topological beams

## Abstract

We report an entoptic phenomenon in which the classical two-lobed Boehm’s brushes are transformed into a multilobed structure by projecting spin–orbit-coupled light onto the human retina. These structured beams, composed of nonseparable superpositions of circular polarization and orbital angular momentum, produce azimuthally modulated entoptic patterns through polarization-dependent scattering in the retina. Unlike Haidinger’s brushes, which arise from dichroic absorption in the macula, the observed effect is driven by angular variations in scattering strength relative to the local polarization direction. In regions where scattering centers exhibit polarization orientations that converge toward a common point, their combined contributions reinforce one another, producing brighter and more sharply defined entoptic lobes whose number and orientation vary systematically with the topology of the spin–orbit stimulus. Psychophysical measurements across retinal eccentricities from 0.5^°^ to 4^°^ in eleven participants revealed that contrast detection thresholds decreased exponentially with eccentricity, consistent with polarization-sensitive scattering by isotropic structures in the nonfoveal retinal regions. From the psychophysical fits, the mean eccentricity at which the entoptic pattern reached a 50% threshold was $r_{50}=1.03^{{}^{\circ}}$ with a 95% CI of [0.72, 1.34]^°^, indicating that the spin–orbit-induced entoptic structure becomes perceptually robust at approximately 1^°^ retinal eccentricity and that perception improves with increasing retinal eccentricity. Together, these findings demonstrate that spin–orbit light modulates scattering-based visual phenomena in previously unrecognized ways, enabling approaches for probing retinal structure and visual processing using topological features of light.

## Significance

The human eye can perceive subtle polarization patterns in light, revealing structural features of the retina without invasive imaging. We identified a visual phenomenon in which structured light manifesting a spatially varying polarization profile produces multilobed polarization patterns on the retina. These patterns arise from polarization sensitive scattering in the eye’s peripheral layers and depend on the light’s topological structure. By linking the geometry of light to visual perception, this work establishes a class of topologically driven entoptic effects and opens pathways for probing retinal integrity, visual processing, and the interaction between structured light and biological tissue.

Structured light, defined by optical fields with tailored amplitude, phase, and polarization profiles, has opened new frontiers in imaging, communication, and quantum information (1–4). Among the most versatile classes of structured beams are those carrying orbital angular momentum (OAM), whose helical wavefronts enable control over light’s spatial structure at both classical and quantum levels (5–11). When OAM is coupled to polarization, the resulting spin–orbit beams exhibit space-varying polarization topologies and nonseparable vectorial modes that can encode rich geometric and topological information (12–14).

The human visual system, though largely insensitive to polarization under normal viewing conditions, is capable of perceiving subtle polarization-induced entoptic phenomena (15–17). The best-known example is Haidinger’s brushes, which are a faint, hourglass-shaped pattern visible in central vision when viewing linearly polarized light that includes blue wavelengths (18–20). It is thought to arise from dichroic absorption by the aligned macular pigment molecules in a radially symmetric Henle fiber layer (21–24). Another lesser-known but distinct phenomenon is Boehm’s brushes (16, 25), which appear as a bowtie-shaped pattern in peripheral vision when a small, linearly polarized point source is viewed in the periphery.

Unlike Haidinger’s brushes, which arise from dichroic absorption in the macula, Boehm’s brushes are attributed to polarization-sensitive scattering from subcellular structures within the inner retina, particularly in layers such as the inner plexiform layer and ganglion cell layer (15, 16, 26, 27).

Classical entoptic patterns have historically been limited to uniform or linearly polarized fields. Recent work has shown that spin–orbit structured light can produce entoptic patterns with azimuthal lobes whose geometry encodes the light’s polarization topology (28–31). These stimuli have been used to modulate Haidinger’s brushes and quantify perceptual thresholds in the macula, enabling discrimination of OAM states and enhancing visibility through spatially varying polarization (32). More recently, structured light has been applied to selectively characterize circularly oriented macular pigment (33), yielding eccentricity-dependent models of optical density based on threshold detection of rotating entoptic patterns. However, these effects have thus far been limited to central, absorption-based mechanisms. Whether polarization topology can also drive entoptic phenomena through peripheral scattering remained unknown, motivating the present study of Boehm’s brushes under structured-light illumination.

In this work, we report the identification of an entoptic phenomenon in which the classical two-lobed Boehm’s brushes are expanded into a multilobed pattern by projecting spin–orbit beams with varying OAM onto the retina. The number and spatial distribution of lobes depend on the OAM difference between the right and left circularly polarized components (Δℓ): When Δℓ<2, the modulation appears outside the stimulus ring, while for Δℓ>2, it shifts inward. Through psychophysical measurements across retinal eccentricities from 0.5^°^ to 4^°^, we found that perceptual thresholds decrease (improve) with increasing eccentricity, consistent with a scattering-based mechanism involving isotropically distributed retinal structures. Our results reveal a previously untapped class of topologically structured entoptic phenomena and suggest tools for noninvasive retinal diagnostics, precision assessment of peripheral polarization sensitivity, and controlled studies of light–matter coupling in human vision.

## 1. Polarization-Sensitive Scattering in the Retina

**Fig. 1.**
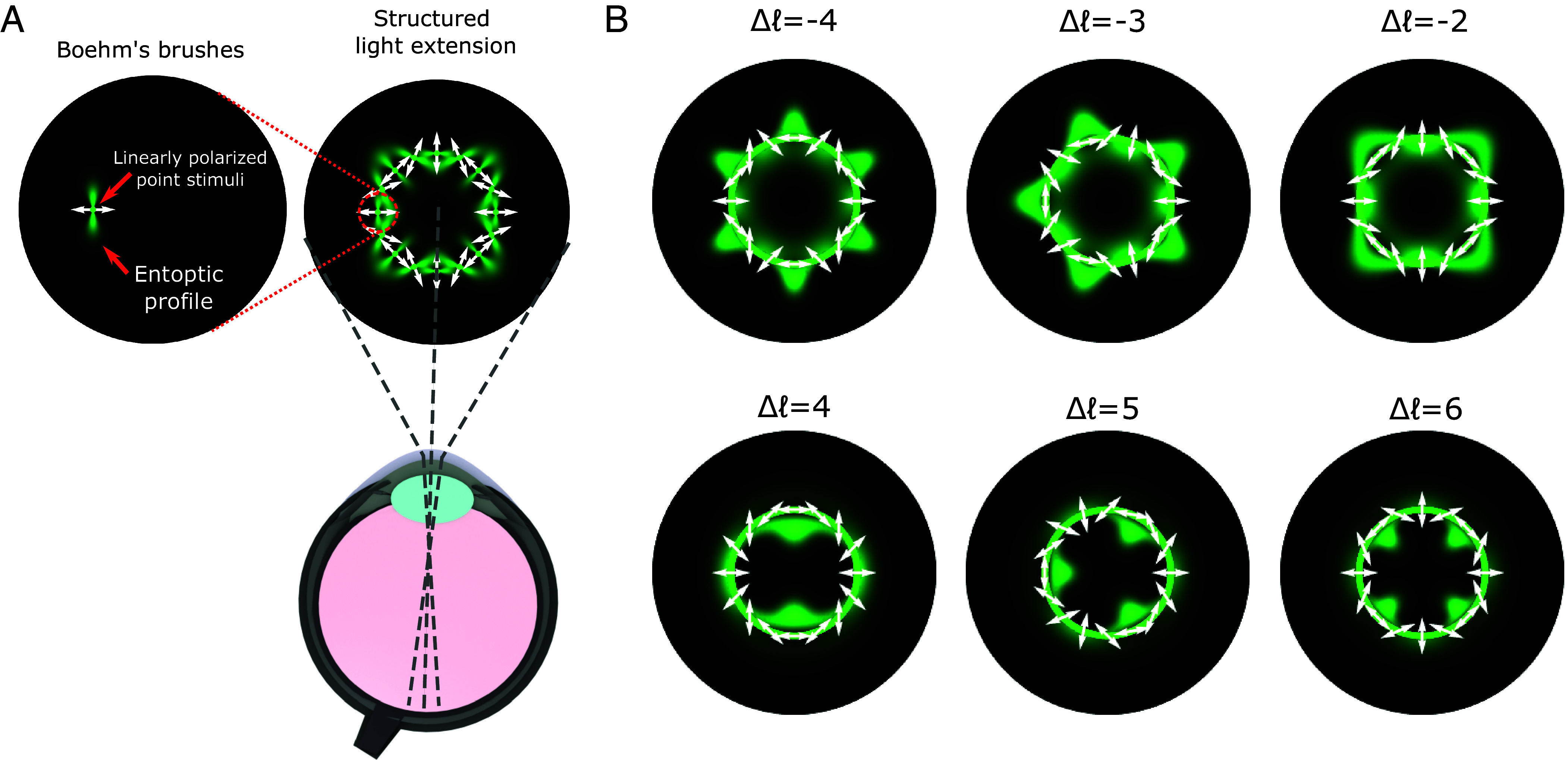
(*A*) Conceptual illustration of how a structured entoptic pattern emerges from spin–orbit light. In this example the relative azimuthal phase winding between the two polarization components is Δℓ=−2. For illustrative clarity, the stimulus is shown here as a discrete ring of points, each with a polarization orientation determined by the local structure of the spin–orbit state, see Eq. **2**. Each point elicits Boehm’s brushes through polarization-sensitive scattering (Eq. **1**), and the perceived global pattern arises from the incoherent sum of these local responses. (*B*) Simulated entoptic profiles corresponding to structured beams with Δℓ ranging from −4 to +6, viewed from an annular (ring-shaped) stimulus. The number of visible lobes follows N=|Δℓ−2|. For Δℓ<2, the polarization modulation is pronounced outside the aperture while for Δℓ>2 the modulation shifts inward, and the structured pattern appears within the dark central region. The annular aperture profile allows access to the inner polarization-dependent scattering response that is otherwise masked by the high central intensity of a disk stimulus.

Entoptic phenomena such as Haidinger’s and Boehm’s brushes arise from interactions between polarized light and structures within the human retina. Haidinger’s brushes, visible in central vision, are attributed to the dichroic absorption of short-wavelength light by macular pigments bound perpendicularly to radially oriented Henle fibers. In contrast, Boehm’s brushes appear as faint patterns outside a small, centrally fixated, polarized point source (Fig. 1*A*) and are thought to result from polarization-sensitive scattering within the layered microstructure of the retina.

While Mie scattering within the retina is the underlying physical mechanism, a rigorous quantitative model linking scattering anisotropy and polarization to the perceived entoptic pattern has yet to be established. For the present analysis, we therefore adopt a phenomenological description that captures the observed spatial form of Boehm’s brushes, centered at a retinal location $\left(x_{0},y_{0}\right)$ away from the central point of vision:

[1] $$\begin{array}{cc} I(x,y)\propto & \exp\left[-\frac{{\left(x-x_{0}\right)}^{2}+{\left(y-y_{0}\right)}^{2}}{2\sigma_{1}^{2}}\right]\\ & \times \exp\left[-\frac{1}{2\sigma_{2}^{2}}\cos^{2}\left(\alpha [x,y]-\theta \right)\right], \end{array}$$

where $\alpha \left[x,y\right]=\;\tan^{-1}[\left(y-y_{0}\right)/\left(x-x_{0}\right)]$, $\sigma_{1}$ characterizes the radial extent, $\sigma_{2}$ the angular sharpness of the lobes, and *θ* is the polarization orientation. In our annular-aperture geometry, the observed pattern corresponds to an azimuthal superposition of such displaced contributions at fixed radius $r_{0}$. That is, the measured intensity can be interpreted as an incoherent sum of responses centered at $\left(r_{0},\phi \right)$ for all *ϕ*, reflecting uniform sampling of the annulus. Here $\left(r_{0},\phi \right)$ denote the polar coordinates of the center $\left(x_{0},y_{0}\right)$, with $x_{0}=r_{0}\cos\phi$ and $y_{0}=r_{0}\sin\phi$.

**Fig. 2.**
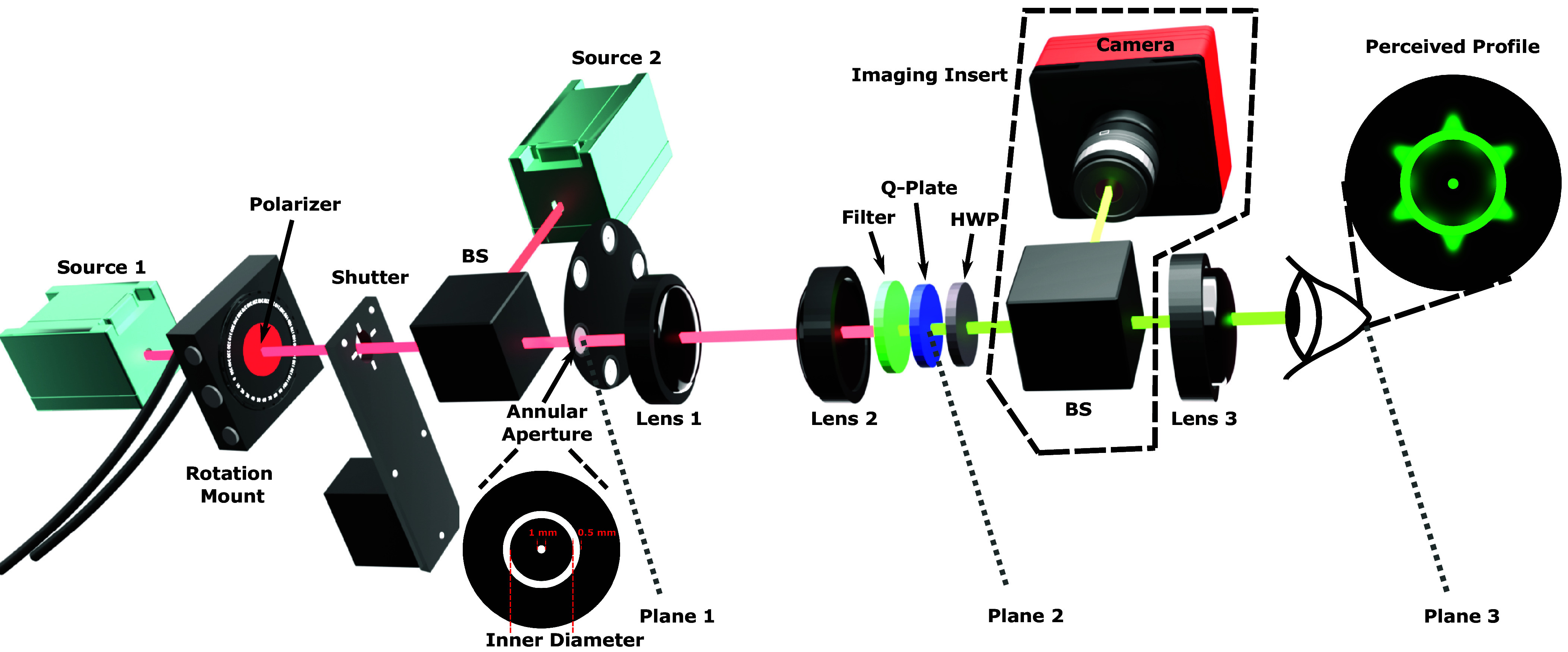
Experimental setup for projecting structured stimuli onto the retina. Two broadband white light sources were combined at a beamsplitter (BS); one arm included a rotating polarizer and shutter, while the other served as a DC offset. This allowed modulation of polarization contrast at fixed intensity. The combined beam passed through an annular aperture and was imaged onto a Q-plate that was followed by a half-wave plate (HWP). This arrangement prepared a spin–orbit beam that induced N=6 outer lobes when perceived by an observer, as shown. The Q-plate output was imaged onto the retina using a f=125 mm lens placed in front of the eye to remove propagation effects. An imaging insert before the final lens enabled alignment with fundus photos to calibrate aperture radius to retinal eccentricity. A 530 nm filter set the stimulus wavelength.

In our experiments, spin–orbit structured light was used to drive this scattering-mediated perceptual channel. These structured-light beams combine circular polarization with OAM, giving rise to azimuthally varying polarization topologies, see Fig. 1*B*. The transverse wavefunction of a spin–orbit beam that is imaged on the retina can be written in the form:

[2] $$\begin{array}{c} \left|\Psi \right\rangle =f\left(r\right)\left[e^{i\Delta \ell \phi }\left|R\right\rangle +\left|L\right\rangle \right], \end{array}$$

where f(r) describes the profile of the stimulus aperture, which in our experiments was an annular profile, see Fig. 2; $\left|R\right\rangle =\left(\begin{array}{c} 1\\ 0 \end{array}\right)$ and $\left|L\right\rangle =\left(\begin{array}{c} 0\\ 1 \end{array}\right)$ denote right- and left-circular polarization states, respectively, and Δℓ is the OAM difference between the right and left circularly polarized states.

The perceived pattern corresponds to the incoherent superposition of locally polarized scattering responses (individual Boehm’s brushes) oriented by the spatially varying polarization of Eq. Eq. **2**. This yields a global entoptic structure with azimuthal modulation, producing a multilobed pattern. The resulting percepts, shown in Fig. 1*B*, feature a number of bright lobes determined by:

[3] $$\begin{array}{c} N=|\Delta \ell -2|, \end{array}$$

reflecting the topological structure of the polarization field. Interestingly, the same lobe-counting relation is observed in previous reports of spin–orbit-modulated Haidinger’s brushes (28).

A notable and unexpected feature of this phenomenon is that the entoptic lobes can appear either inside or outside the visible stimulus region. For Δℓ<2, the constructive intensity amplification is away from the stimuli, resulting in outer lobes. Conversely, for Δℓ>2, the constructive intensity leads to lobes perceived within the inner region of the annular stimulus. This behavior is illustrated in Fig. 1*B*, which shows how the apparent lobe location depends on Δℓ. The perceived lobe geometry is invariant under changes in eye position: Shifting fixation does not alter the number or orientation of lobes. However, the perceived contrast varies across the retina, reflecting local differences in the density and scattering properties of subcellular structures.

## 2. Methods

### 2.1. Experimental Setup.

The experimental system, depicted in Fig. 2, was designed to deliver spin–orbit-coupled light beams with tunable spatial structure to the human retina via an annular aperture. All optical components were aligned to maintain polarization fidelity and spatial mode purity at the retina of the eye. The optical stimulus was generated using two intensity-variable white light sources directed into a nonpolarizing 50:50 beamsplitter. One input arm contained a rotating linear polarizer and delivered a polarized beam, while the other arm delivered an unpolarized beam. By balancing the relative intensities of the polarized and unpolarized components, the contrast of the structured stimulus was precisely controlled while keeping the total light intensity constant. A mechanical shutter after the polarizer was used to define the stimulus presentation window, ensuring a precise 250 ms exposure time independent of the motor’s acceleration or deceleration phases.

The importance of rotation speed was established in preliminary trials, which showed that faster or slower spin–orbit beam rotation effectively stretched or compressed the threshold-eccentricity curve. Based on pilot data, polarizer rotation speed 540^°^/s was chosen to capture the contrast threshold decay behavior in typical participants. Note that every 180^°^/s rotation of the polarizer corresponds to one full period of rotation for all entoptic patterns depicted in Fig. 1*B*. For the six-lobed structure shown in Fig. 2, this represents a physical rotation of 60^°^/s; therefore, a polarizer rotation speed of 540^°^/s corresponds to an effective 180^°^/s physical rotation of the perceived pattern.

The output of the beamsplitter was directed through an interchangeable annular aperture. Six annular apertures were used, each with a 0.5 mm-wide opening and inner diameters of 3.9, 7.1, 10.3, 13.5, 16.7, and 19.9 mm. For data analysis, the corresponding retinal eccentricities were defined by the midpoint radius of each annulus, i.e., the average of its inner and outer edges. The geometry of the annular aperture is illustrated in Fig. 2. Retinal eccentricity is the angular distance (in degrees) of a visual stimulus from the point of direct gaze. It measures the distance between the center of the fovea and the image on the retina. More simply: How far an image of a point seen in the visual field lies in the peripheral retina. A separate 1 mm central circular opening served as the fixation point and was covered with transparent tape to depolarize the light in that region.

A narrowband optical filter centered at 530 nm with a full width at half maximum (FWHM) of 10 nm was placed before the Q-plate to ensure a well-defined wavelength, selected for its high perceptual sensitivity to polarization-dependent scattering (27) and minimal macular pigment absorption associated with the visibility of the Haidinger’s brushes phenomenon (34).

The aperture plane (Plane 1) was imaged onto a Q-plate using a 4f imaging system consisting of two lenses each with a focal distance of 100 mm. The Q-plate is a liquid crystal optical element that can form a light beam with OAM given a polarized input (14). In our experiment we used a Q-plate with topological charge q=1 optimized for 532 nm light. For a linearly polarized input, the output state is an equal superposition of opposite circular polarizations carrying opposite OAM,

[4] $$\begin{array}{c} |\Psi_{Q}\rangle =\frac{1}{\sqrt{2}}\left(e^{i\ell \phi }\left|R\right\rangle +e^{-i\ell \phi }\left|L\right\rangle \right), \end{array}$$

where ℓ=2q=2.

The state at the output plane of the Q-plate (Plane 2) is imaged onto the retina using a lens of focal length f=125 mm placed directly in front of the eye. This imaging configuration maps the transverse state profile without additional propagation-induced phase evolution, so the relevant quantity for retinal response is the local polarization state, thereby allowing us to factor out a common phase and consider the state:

[5] $$\begin{array}{c} |\Psi_{\text{ret}}\rangle =\frac{1}{\sqrt{2}}\left(e^{i\Delta \ell \phi }\left|R\right\rangle +\left|L\right\rangle \right), \end{array}$$

where Δℓ=4 is the relative azimuthal phase winding between the two polarization components. The three-lens system was chosen such that the annular apertures correspond to retinal eccentricities in the range 0.5^°^ to 4^°^.

A half-wave plate placed after the Q-plate flips the circular polarization components, |R⟩↔|L⟩, thereby exchanging the OAM carried by each component. This adjustment effectively changed Δℓ from 4 to −4, resulting in an entoptic pattern with six outer azimuthal lobes instead of two inner lobes (Eq. **3**).

To accurately calibrate the visual eccentricity of each annular aperture, an imaging insert was incorporated into the setup. This insert consisted of a beamsplitter, an imaging lens, and a camera, enabling retinal imaging under illumination by the setup. Retinal images were acquired through the camera system and later compared to participant-specific fundus photographs, enabling extraction of the annular aperture radius-to-retinal eccentricity conversion for each individual.

### 2.2. Participants.

All participants were recruited at the University of Waterloo. Participants provided written informed consent to take part in the study. The study protocol was approved by the institutional ethics committee at the University of Waterloo, in accordance with the Declaration of Helsinki. All participants were naive to the experimental hypothesis and remunerated for their time. All tests were performed on the right eye only. Clinical screening included: habitual visual acuity using an Early Treatment of Diabetic Retinopathy Study (ETDRS) chart calibrated for 4 m, objective refractive error determined by an autorefractor (Topcon KR-1, Tokyo, Japan), structural macular integrity by optical coherence tomography (Topcon 3D OCT-1, Tokyo, Japan) and macular luminance sensitivity by microperimetry (4 to 2 (fast) threshold strategy for central 8 degrees; Nidek MP-3, Aichi, Japan). Fundus imaging was performed undilated with the Nidek MP-3. Participants were excluded if they had visual acuity poorer than 0.2 logMAR, any abnormality visible on macular OCT or an absolute loss of sensitivity on any tested point on microperimetry.

### 2.3. Psychophysical Procedure.

Participants completed two structured-light testing sessions following routine optometric screening to verify normal or corrected-to-normal visual acuity and binocular alignment. Session 1 included retinal imaging and a familiarization task to assess perceptual visibility of the structured stimulus. Retinal images were acquired using the same optical system equipped with an imaging insert, capturing views through a calibration aperture. These images were compared with fundus photographs to map aperture geometry to visual angle, allowing subject-specific linkage between annular aperture size and retinal eccentricity.

The familiarization task presented high-contrast clockwise and counterclockwise rotating stimuli. Participants then completed ten randomized trials at fixed contrast to verify comprehension of the task and alignment within the apparatus. Only those achieving at least 8 out of 10 correct responses were advanced to the main contrast-thresholding task.

Session 2 involved quantifying perceptual sensitivity using a two-alternative forced-choice (2AFC) contrast detection task with a 2-up/1-down staircase procedure, converging at 70.7% accuracy. A detailed description of the protocol for session 2 is given in *SI Appendix*. Each annular aperture condition was tested using two interleaved staircases: Staircase A began at a contrast of 80%, and Staircase B at 30%. Each staircase consisted of 14 reversals (maximum 90 trials), with step sizes decreasing across four stages: 0 to 2 reversals, 0.1; 3 to 5 reversals, 0.05; 6 to 8 reversals, 0.025; and 9 to 14 reversals, 0.0125. Individual contrast thresholds were computed as the arithmetic mean of the final six reversal points.

The structured-light stimulus was presented for 250 ms, after which the polarized light path was closed. Participants then reported the perceived direction of azimuthal rotation (clockwise or counterclockwise). Contrast decreased following two consecutive correct responses and increased after a single incorrect response. The response to each trial triggered the onset of the next one. Between aperture conditions, participants were offered a short break lasting a few minutes.

The full protocol consisted of six randomized aperture conditions, corresponding approximately to retinal eccentricities between 0.5^°^ and 4^°^, with presentation order counterbalanced across participants. These eccentricities were selected because Boehm’s brushes are typically absent at the fovea and most prominent around 4^°^, making this range optimal for probing its perceptual onset and peripheral enhancement.

**Fig. 3.**
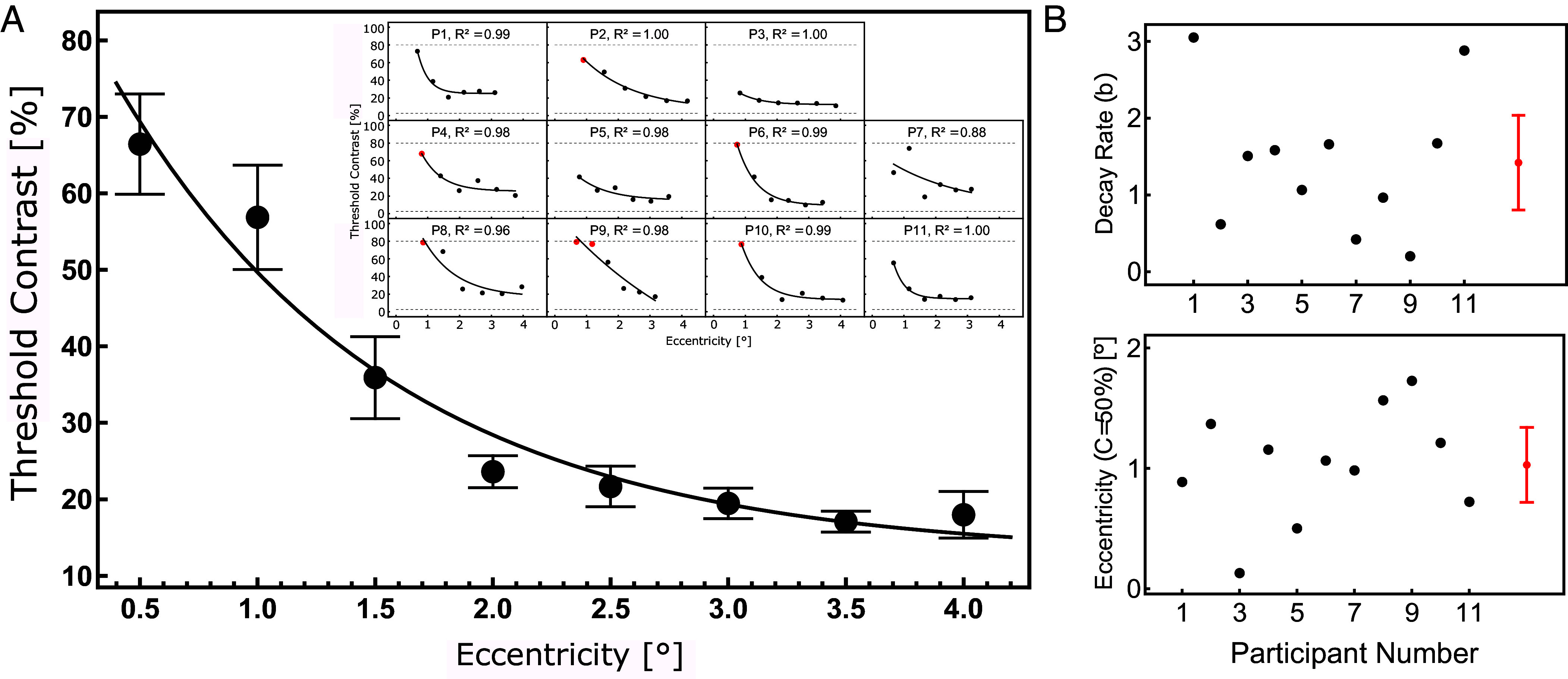
(*A*) Group-averaged contrast thresholds for entoptic pattern detection across retinal eccentricity. Black points indicate the mean threshold across participants (11 total), where each point represents the average of data within eccentricity bins centered at 0.5^°^, 1.0^°^, 1.5^°^, 2.0^°^, 2.5^°^, 3.0^°^, 3.5^°^, and 4^°^ with a bin width of ±0.25^°^. The solid curve shows the best-fit exponential function, $T\left(r\right)=0.13+0.87e^{-0.85r}$, which captures the group-averaged decay in contrast threshold with increasing eccentricity. The *Inset* shows the individual participant data and fits, illustrating the consistency of the exponential decay across subjects. The red points denote instances where the staircase reached the ceiling (maximum contrast) in two or more of the last six reversals. Note that these represent underestimates of the true threshold. The stimulus contrast range in the setup spanned from 80% to 2.5%. (*B*) Summary of fitted model parameters across participants. The *Upper* panel shows the distribution of decay constants (*b*) extracted from the individual fits, with a group mean of $b=1.42\deg^{-1}$ and a 95% CI of [0.80, 2.04]$\deg^{-1}$. The *Lower* panel shows the eccentricity at which the fitted models reached a contrast threshold of 50%, with a mean value of $r_{50}=1.03^{{}^{\circ}}$ and a 95% CI of [0.72, 1.34]^°^. These results indicate that the entoptic polarization pattern becomes perceptually robust at approximately 1^°^ retinal eccentricity. Across all participants, thresholds were highest near the fovea and decreased (improved) with increasing retinal eccentricity, consistent with a polarization-sensitive scattering mechanism in the retina.

Sixteen participants were initially enrolled. One participant withdrew from the study before scheduling Session 2, and another was found ineligible during the familiarization task of Session 2. Two participants were unable to complete the structured-light retinal imaging due to technical difficulties and did not proceed to Session 2. One additional participant completed both sessions but expressed discomfort and exhibited no measurable sensitivity, with all thresholds approaching the maximum contrast limit. This participant was excluded from further analysis. The remaining eleven participants successfully completed the full protocol and were included in the results shown in Fig. 3.

## 3. Results and Discussion

Contrast detection thresholds consistently decreased (improved) with increasing retinal eccentricity, see Fig. 3. Visual inspection of the data in Fig. 3*A* revealed a steep initial decline in contrast threshold near the fovea followed by a gradual asymptote at larger eccentricities. This pattern is characteristic of an exponential decay and therefore we can model each participant’s data using the function

[6] $$\begin{array}{c} T\left(r\right)=ae^{-br}+c, \end{array}$$

where T(r) is the contrast threshold at eccentricity *r*, and a,b,c are fit parameters. Each participant’s data were fit individually using this model, yielding high coefficients of determination (R^2^) across all subjects (*Inset* of Fig. 3*A*), confirming that the exponential function captures the observed relationship between threshold and eccentricity.

Fig. 3*A* shows the group-averaged thresholds, obtained by binning data within eccentricity intervals centered at 0.5^°^, 1.0^°^, 1.5^°^, 2.0^°^, 2.5^°^, 3.0^°^, 3.5^°^, and 4^°^ (bin width ±0.25^°^). The solid curve represents the best-fit exponential to these binned data, $T\left(r\right)=0.13+0.87\;e^{-0.85\;r}$, which reproduces the overall decline in threshold with increasing eccentricity. The *Inset* illustrates individual fits for all participants, demonstrating consistent exponential behavior across subjects.

The distribution of fitted decay constants (*b*) across participants is shown in the *Top* panel of Fig. 3*B*, with a mean value of $b=1.42\;{\text{deg}}^{-1}$ and a 95% CI of [0.80, 2.04]${\text{deg}}^{-1}$. The *Lower* panel shows the eccentricities at which the fitted curves reached a 50% contrast threshold, with a mean value of $r_{50}=1.03^{{}^{\circ}}$ and a 95% CI of [0.72, 1.34]^°^. These results indicate that the entoptic pattern becomes perceptually robust at approximately 1^°^ retinal eccentricity.

While individual thresholds varied, all participants exhibited the same qualitative decay behavior, supporting a common underlying mechanism. Variation in absolute sensitivity may reflect anatomical differences in the density, distribution, or optical properties of retinal scatterers. The consistent decline in threshold with eccentricity supports the conclusion that this structured entoptic phenomenon arises from peripheral, polarization-dependent scattering distinct from macula-confined absorption-based effects such as Haidinger’s brushes.

This behavior is consistent with classical measurements of Boehm’s brushes under uniform linear polarization. In particular, Vos and Bouman (26) reported low sensitivity near the fovea with a gradual increase toward a peak at larger eccentricities (4.5 to 5^°^). Our observed decrease in detection threshold over the range 0 to 4^°^ therefore corresponds to the rising portion of this established sensitivity curve.

## 4. Conclusion

We have shown that structured light carrying spin–orbit coupling elicits a previously unreported entoptic phenomenon in which the classical two-lobed Boehm’s brushes expand into a multilobed pattern. The appearance and geometry of these lobes arise from polarization-sensitive scattering in the retina and vary systematically with the topological charge and polarization structure of the incident beam. Psychophysical measurements revealed an exponential decline in detection threshold with increasing retinal eccentricity, with the entoptic response becoming robustly visible near 1^°^ eccentricity. Together, these results demonstrate that topologically structured polarization fields can directly modulate scattering-based entoptic perception, providing an experimental route for screening retinal integrity, probing peripheral visual sensitivity, and investigating the interaction between structured light and human vision.

Possible future directions include developing quantitative models of the entoptic response using contrast sensitivity functions adapted for polarization-dependent scattering, analogous to those formulated for absorption-based entoptic phenomena (33, 35). Such a model would enable extraction of an effective optical density of the polarization-sensitive scattering centers from the measured contrast thresholds, providing a quantitative link between entoptic perception and the underlying retinal microstructure. The results could be compared with OCT-derived retinal thickness profiles to evaluate potential correlations between perceptual thresholds and retinal micro- structure. Comparative studies between healthy and clinically affected populations could reveal how diseases such as early-stage age-related macular degeneration and diabetic maculopathy alter the spatial distribution and effective optical properties of polarization-sensitive scattering structures. In glaucoma, early perimacular loss of the retinal nerve fiber layer may modify scattering dynamics, potentially leading to detectable changes in entoptic perception and enabling functional assessment of disease onset and progression.

## Supplementary Material

Appendix 01 (PDF)

## Data Availability

All study data are included in the article and/or *SI Appendix*.
