# Supplementary material for: Topological expansion of Boehm’s brushes via structured light
Source: Proc Natl Acad Sci U S A. 2026 Jul 9;123(28):e2532243123. doi: 10.1073/pnas.2532243123 (PMC13367876; doi:10.1073/pnas.2532243123)
Supplement: Supplementary file 1 — Appendix 01 (PDF) [file pnas.2532243123.sapp.pdf]

Supporting Information for

Topological Expansion of Boehm's Brushes via Structured Light

D. A. Pushin, I. Salehi, A. Chow, A. E. Silva, P. Chahal, D. G. Cory, M. Kulmaganbetov, G. P. Misson, N. Shentevski, T. Singh, S. E. Temple, B. Thompson, and D. Sarenac

D. Sarenac.
E-mail: dusansar@buffalo.edu

This PDF file includes:

Supporting text
Fig. S1

Supporting Information Text

1. Experimental Protocol

The entire experiment was controlled by a custom Python program running on Windows, which handled all stages of the procedure including loading calibration data, implementing staircase logic, and automatically saving participant output to ID-labeled folders. All optomechanical components were sourced from a single manufacturer (Thorlabs Inc.) to ensure full integration and compatibility, and the Pylablib package was used to interface with Thorlabs devices through Python.

For each participant, testing began with the selection of an annular aperture according to a predefined, counterbalanced order covering six conditions. Once the aperture was set, both light paths were activated to generate a rotating spin–orbit stimulus at 80 % contrast. This high-contrast rotating pattern served as a brief preparation phase prior to the first trial of each aperture condition.

After the participant confirmed proper head alignment with the optical axis, the test was initiated by pressing the central key on a three-button keypad. This triggered closure of the mechanical shutter blocking the polarized light path. The program then randomly selected one of two interleaved staircases: Staircase A, beginning at 80 % contrast, or Staircase B, beginning at 30 %. For each subsequent trial, contrast levels were adjusted dynamically based on the history of the selected staircase.

Once the contrast was set by modulating the current to the two light sources, the motor accelerated to the target rotation speed of 540°/s. The shutter then opened for a 250 ms exposure and closed immediately afterward, halting the rotation. Participants indicated the perceived rotation direction using the keypad (right = clockwise, left = counterclockwise).

The program evaluated the response, logged its correctness, updated the reversal status, and recorded all relevant data, including staircase history. After each trial, termination criteria were checked; if met, the current staircase was concluded. The program then selected the next staircase at random and repeated the process. Upon completion of both staircases for a given aperture, the system automatically advanced to the next aperture and continued until all six conditions were completed.

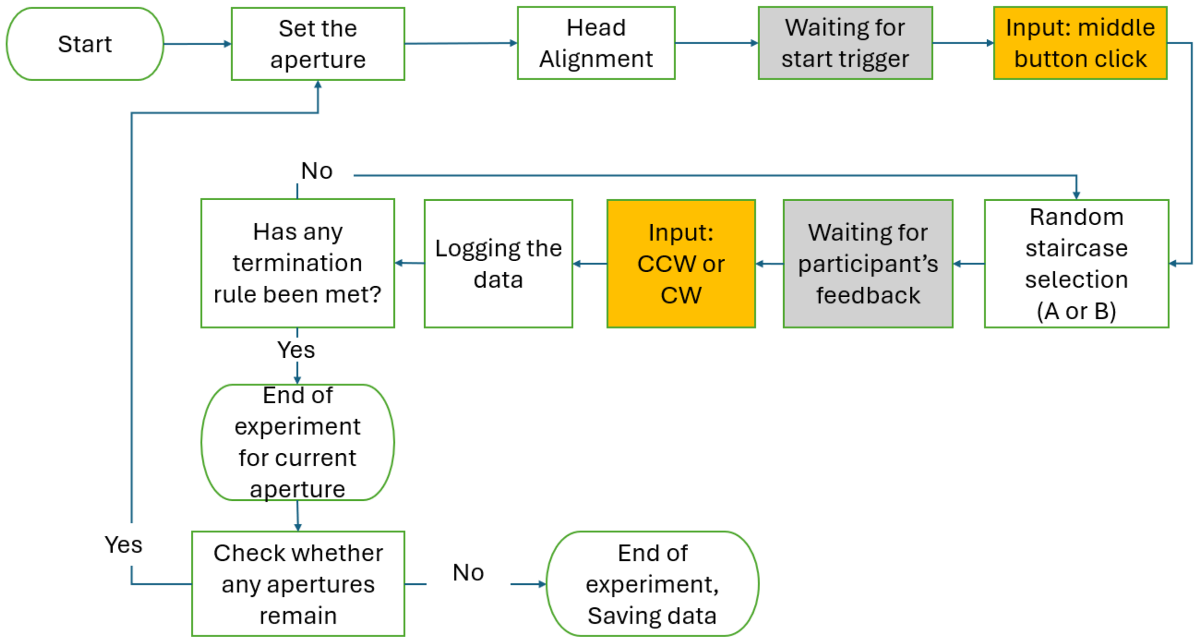

Fig. S1. Flowchart of the fully automated experimental procedure. A Python program controlled all aspects of the task, including loading calibration data, setting aperture order, managing the rotating spin–orbit stimulus, implementing the interleaved staircases, and logging responses. Participants initiated each trial and provided feedback via a three-button keypad (right button = clockwise, left button = counterclockwise), while all other steps—including contrast adjustment, stimulus presentation, and progression through apertures—were executed automatically by the system.
